# 

**Published:** 1922-06

**Authors:** 


### HOSPITAL and HEALTH

REVIEW

*Established 1886* *No.* 1851. *Vol. LXXI.*

*New Series.* Vol. 1. No. 9 *June* 1922 Price Sixpence

CONTENTS.

PAGE

NOTES AND COMMENTS :—

*Health.*—Anti-Epidemic Work in Eastern Europe —Wellington House, 1911-1922—The School of Hygiene—The Success of Infant Welfare Work—The National Council of Mental Hygiene—The Welfare of the Blind—The Problem of the Typhoid "Carrier"—The Causation of Miners' Blindness —The Superannuation Bill—The School Medical Services—Safety First—Child Welfare in Denmark 241

*Hospitals.*—Allegations Against Asylum Attendants—The Labour Party and the Hospitals—The British Hospitals Association Conference—The Combined Appeal—The Hospital Procession —A Dispenser's Fatal Mistake—Smoking Rooms in Nurses' Homes—The Removal of a Burden—Is Water Wasted in Hospitals? . . . . . . 246

THE CHANGE IN HOSPITAL FINANCE . . . . 249
By Sir Alan Garrett Anderson, K.B.E.

THE GREAT DROUGHT OF 1921: CHLORINATION OF IMPURE WATER SUPPLIES . . . . . . . 253

By Sir Alexander C. Houston, K.B.E., M.B., D.Sc.

PAGE

THE EFFECTS OF THE DROUGHT OF 1921 ON WATER SUPPLY . . . . . . . . . . . 255

THE PUBLIC HEALTH: DOES IT MATTER? . . . . 257

THE DECLINE AND FALL (?) OF TUBERCULOSIS.—III. . 258
By Edgar L. Collis, M.A., M.D., M.R.C.P.

REVIEWS . . . . . . . . . . 262

HOSPITAL FINANCE . . . . . . . . 264

HOSPITAL NEWS . . . . . . . . . 266

CORRESPONDENCE :—

The British Medical Association and the Voluntary Hospitals (N. Bishop Harman, F.R.C.S.)—Advertising a Contribution Scheme (S. R. Lamb) —Approved Societies and Hospital Treatment (Sir James Michelli, F. Squires)—The Treatment of Tonsils and Adenoids (Thos. Clapham) . . 268

APPOINTMENTS . . . . . . . . 270

PARLIAMENTARY NEWS . . . . . . . 271

OFFICIAL PUBLICATIONS . . . . . . . 272

